# The Potential of Food By-Products: Bioprocessing, Bioactive Compounds Extraction and Functional Ingredients Utilization

**DOI:** 10.3390/foods11244092

**Published:** 2022-12-17

**Authors:** Michela Verni, Federico Casanova

**Affiliations:** 1 Department of Soil, Plant, and Food Sciences, University of Bari “Aldo Moro”, Via Amendola 165/A, 70126 Bari, Italy; 2 Research Group for Food Production Engineering, National Food Institute, Technical University of Denmark, Søltofts Plads, 2800 Kongens Lyngby, Denmark

Achieving sustainability in the agro-food sector can only be possible with the valorization of food industry waste and side streams, products with an extremely high intrinsic value but often discarded because they are unfit for further processing that meets consumer expectations. Apart from their use as feed, a more practical solution responding to the modern vision of a circular economy must be sought. In this framework, this Special Issue aimed at covering the most recent advances in the valorization of food by-products (of both animal and plant origin), from extraction to bioprocessing, including their application in a variety of food-related industries throughout the entire food supply chain.

Several processes can guarantee that the potential of food industry by-products is fully unleashed, generating high-value ingredients while ensuring nutritional quality. For example, ultrasound technology was successfully used to extract phenolic acids, flavonoids, anthocyanins, and carotenoids from *Morus alba* leaves [1], pomegranate peels and seeds [2], guaraná by-products [3], and horchata, a Spanish beverage obtained from pressing tiger nut by-products [4]. Such side streams, rich in phenolic compounds, are often studied for their beneficial properties on human health, particularly their antioxidant properties determined in vitro [1,2,4,5,6]. The extraction of olive pomace phenolic compounds, followed by their purification using macroporous absorbing resins was also explored by Zhao et al. [5], confirming the great potential as natural antioxidants, preservatives, and antimicrobials in clean-label foods.

Enzymatic treatments, alone or ultrasound-assisted, are among the processes employed to valorize food industry waste and by-products [7,8,9]. Ultrasound-assisted enzymatic treatment was often used to extract soluble and insoluble dietary fibers from orange peel [7] and olive pomace [8]. The renowned physiological effects of dietary fibers are varied and often inversely correlated to obesity, type two diabetes, cancer, and cardiovascular disease. Lignocellulosic-based adsorbents from sugarcane bagasse, cornstalk piths, and corn cob were also found to improve the physicochemical properties and quality of fried oils [10]; nevertheless, beyond the technological properties of dietary fibers, their cholesterol adsorption capacity [7] and antioxidant and prebiotic activity [8] suggest that the application of such by-products in functional foods should be recommended. Indeed, L-arabinose, a bio-active compound derived from the processing of many fibrous materials, was studied by Pol et al. [11], who concluded that its addition to sugary drinks used for a clinical trial, despite the large quantities of starch and fat, significantly lowered postprandial glycemic and insulinemic responses in healthy subjects.

Although the sole extraction and characterization of the compounds of interest is the most preferred option, as in the above-reported cases, as well as for *Aloe vera* anthraquinones [12] or sesame oil asarin [13], the use of the whole discarded biomass is the valorization alternative that can prevent the further generation of by-products and should be preferred in terms of sustainability of the overall upcycling process. This option was chosen by Najjar et al. [14] for the valorization of date seed flour, incorporating it in composite cookies and thus leading to a high amount of total polyphenolic content, with flavonoids showing high in vitro antioxidant activity.

Two review articles were also collected in this Special Issue. One of them overviewed the development of starch-based films as environmentally friendly packaging alternatives which could also be prepared by adding antioxidant or anti-bacterial substances, thus extending food shelf-life, and reducing food waste [15]. The other review focused on the interaction of dairy and plant-based proteins, more specifically, the application of pulsed electric field technology to enhance the functional properties of food proteins [16]. Indeed, the colloidal and acid gelling properties of mixed suspensions of pea and milk proteins [17], and the foaming properties of pea protein isolates and fish skin gelatin [18] were the subject of other studies. In addition, Guan et al. [19] exploited the use of aquatic collagen, obtained from by-products of fish processing, concluding that its higher thermal stability compared to that of terrestrial sources of collagen provides promising applications in food, cosmetics, and biomedical fields.

The research papers published in this Special Issue represent some of the novel strategies at our disposal to valorize waste and by-products generated by the food industry. However, much more support is needed in the future, from academia, industry, government, and consumers, to make sure more sustainable approaches can be put in place to minimize or counteract this issue.

## References

[1] Martín-García B., Aznar-Ramos M., Verardo V., Gómez-Caravaca A. (2022). The Establishment of Ultrasonic-Assisted Extraction for the Recovery of Phenolic Compounds and Evaluation of Their Antioxidant Activity from Morus alba Leaves. Foods.

[2] Campos L., Seixas L., Dias S., Peres A., Veloso A., Henriques M. (2022). Effect of Extraction Method on the Bioactive Composition, Antimicrobial Activity and Phytotoxicity of Pomegranate By-Products. Foods.

[3] Pinho L., de Lima P., de Sá S., Chen D., Campanella O., da Costa Rodrigues C., Favaro-Trindade C., Pinho L.S., de Lima P.M., de Sá S.H.G. (2022). Encapsulation of Rich-Carotenoids Extract from Guaraná (*Paullinia cupana*) Byproduct by a Combination of Spray Drying and Spray Chilling. Foods.

[4] Razola-Díaz M., Gómez-Caravaca A., Guerra-Hernández E., Garcia-Villanova B., Verardo V. (2022). New Advances in the Phenolic Composition of Tiger Nut (*Cyperus esculentus* L.) by-Products. Foods.

[5] Zhao H., Avena-Bustillos R., Wang S. (2022). Extraction, Purification and In Vitro Antioxidant Activity Evaluation of Phenolic Compounds in California Olive Pomace. Foods.

[6] Jiang S., Yu M., Wang Y., Yin W., Jiang P., Qiu B., Qi H. (2022). Traditional Cooking Methods Affect Color, Texture and Bioactive Nutrients of Undaria pinnatifida. Foods.

[7] Sang J., Li L., Wen J., Gu Q., Wu J., Yu Y., Xu Y., Fu M., Lin X. (2021). Evaluation of the Structural, Physicochemical and Functional Properties of Dietary Fiber Extracted from Newhall Navel Orange By-Products. Foods.

[8] Li Y., Yu Y., Wu J., Xu Y., Xiao G., Li L., Liu H. (2022). Comparison the Structural, Physicochemical, and Prebiotic Properties of Litchi Pomace Dietary Fibers before and after Modification. Foods.

[9] Gruppi A., Dermiki M., Spigno G., FitzGerald R. (2022). Impact of Enzymatic Hydrolysis and Heat Inactivation on the Physicochemical Properties of Milk Protein Hydrolysates. Foods.

[10] Ahmed E., Zeitoun A., Hamad G., Zeitoun M., Taha A., Korma S., Esatbeyoglu T. (2022). Lignocellulosic Biomasses from Agricultural Wastes Improved the Quality and Physicochemical Properties of Frying Oils. Foods.

[11] Pol K., Puhlmann M., Mars M. (2022). Efficacy of L-Arabinose in Lowering Glycemic and Insulinemic Responses: The Modifying Effect of Starch and Fat. Foods.

[12] Sadiq U., Gill H., Chandrapala J. (2022). Temperature and pH Stability of Anthraquinones from Native Aloe vera Gel, Spray-Dried and Freeze-Dried Aloe vera Powders during Storage. Foods.

[13] Yu Q., Wang X., Liu H., Ma Y. (2022). Preparation and Characterization of Solid Acid Catalysts for the Conversion of Sesamin into Asarinin in Sesame Oil. Foods.

[14] Najjar Z., Kizhakkayil J., Shakoor H., Platat C., Stathopoulos C., Ranasinghe M. (2022). Antioxidant Potential of Cookies Formulated with Date Seed Powder. Foods.

[15] Liu D., Zhao P., Chen J., Yan Y., Wu Z. (2022). Recent Advances and Applications in Starch for Intelligent Active Food Packaging: A Review. Foods.

[16] Taha A., Casanova F., Šimonis P., Stankevič V., Gomaa M., Stirkė A. (2022). Pulsed Electric Field: Fundamentals and Effects on the Structural and Techno-Functional Properties of Dairy and Plant Proteins. Foods.

[17] Oliveira I., de Paula Ferreira I., Casanova F., Cavallieri A., Lima Nascimento L., de Carvalho A., Nogueira Silva N. (2022). Colloidal and Acid Gelling Properties of Mixed Milk and Pea Protein Suspensions. Foods.

[18] Odelli D., Sarigiannidou K., Soliani A., Marie R., Mohammadifar M., Jessen F., Spigno G., Vall-llosera M., de Carvalho A., Verni M. (2022). Interaction between Fish Skin Gelatin and Pea Protein at Air-Water Interface after Ultrasound Treatment. Foods.

[19] Guan Y., He J., Chen J., Li Y., Zhang X., Zheng Y., Jia L. (2022). Valorization of Fish Processing By-Products: Microstructural, Rheological, Functional, and Properties of Silver Carp Skin Type I Collagen. Foods.

